# Reduced cardiac output in imported *Plasmodium falciparum *malaria

**DOI:** 10.1186/1475-2875-10-160

**Published:** 2011-06-09

**Authors:** Johanna Herr, Parisa Mehrfar, Stefan Schmiedel, Dominic Wichmann, Norbert W Brattig, Gerd D Burchard, Jakob P Cramer

**Affiliations:** 1 University Medical Center Hamburg-Eppendorf, Section Tropical Medicine, Bernhard-Nocht-Strasse 74, 20359 Hamburg, Germany; 2 University Medical Center-Hamburg-Eppendorf, Department Intensive Care Medicine, Martinistr. 52, 20246 Hamburg, Germany; 3 Bernhard Nocht Institute for Tropical Medicine, Department of Molecular Medicine, Bernhard-Nocht-Strasse 74, 20359 Hamburg, Germany; 4 Bernhard Nocht Institute for Tropical Medicine, Clinical Research Group, Bernhard-Nocht-Strasse 74, 20359 Hamburg, Germany

## Abstract

**Background:**

Volume substitution remains subject of controversy in the light of effusions and oedema potentially complicating this highly febrile disease. Understanding the role of myocardial and circulatory function appears to be essential for clinical management. In the present study, cardiac function and cardiac proteins have been assessed and correlated with parasitological and immunologic parameters in patients with imported *Plasmodium falciparum *malaria.

**Methods:**

In a prospective case-control study, 28 patients with uncomplicated and complicated *P. falciparum *malaria were included and findings were compared with 26 healthy controls. Cardiac function parameters were assessed by an innovative non-invasive method based on the re-breathing technique. In addition, cardiac enzymes and pro- and anti-inflammatory cytokines were measured and assessed with respect to clinical symptoms and conditions of malaria.

**Results:**

Cardiac index (CI) as a measurement of cardiac output (CO) was 21% lower in malaria patients than in healthy controls (2.7 l/min/m^2 ^*versus *3.4 l/min/m^2^; P < 0.001). In contrast, systemic vascular resistance index (SVRI) was increased by 29% (32.6 mmHg⋅m^2^/(l/min) versus 23.2 mmHg⋅m^2^/(l/min); P < 0.001). This correlated with increased cardiac proteins in patients versus controls: pro-BNP 139.3 pg/ml versus 60.4 pg/ml (P = 0.03), myoglobin 43.6 μg/l versus 27.8 μg/l (P = < 0.001). All measured cytokines were significantly increased in patients with malaria. CI, SVRI as well as cytokine levels did not correlate with blood parasite density.

**Conclusions:**

The results support previous reports suggesting impaired cardiac function contributing to clinical manifestations in *P. falciparum *malaria. Findings may be relevant for fluid management and should be further explored in endemic regions.

## Background

Circulatory collapse and symptoms associated with impaired hemodynamic function are characteristics of complicated *Plasmodium falciparum *malaria [1]. While impaired cardiac function has long been established as a key component of bacterial sepsis and septic shock, the role of the heart in severe malaria has only recently been started to be further explored. Ehrhardt *et al *have shown that cardiac enzymes are elevated in complicated malaria [2,3]. Ejection fraction assessed by ultrasound in children with severe malaria was significantly reduced on admission compared with discharge [4].

In sepsis, reduced pre-load, myocardial suppression as well as reduced after-load contribute to hyperdynamic but insufficient cardiac function resulting in tachycardia and hypotension. Both, direct toxic effects of bacterial agents as well as excessive production of cytokines and immune mediators have been identified to cause this dysfunction [5-7]. Rapid and abundant rehydration, use of catecholamines for positive inotropic cardiac support as well as peripheral vasoconstriction are well-established therapeutic options. In contrast, some pathomechanisms like parasite sequestration in small vessels and capillary leakage associated with the risk of effusions and oedema are malaria-specific, while other features such as disturbed microcirculation and lactic acidosis as well as excessive production of pro-inflammatory cytokines, are similar in malaria and bacterial sepsis [8,9]. Consequently, there is an ongoing debate on whether and to which extent volume substitution should be recommended in malaria patients [10,11].

Severe malaria is still a leading cause of death especially in children in sub-Saharan Africa. Mortality rates of severe malaria in these regions remain high as anti-parasitic treatment is often insufficient to prevent fatal outcome. Understanding the role of the heart in clinical manifestation of malaria is essential to improve fluid management and symptomatic treatment for this often enough fatal disease.

While biochemical markers and ultrasound findings suggest cardiac impairment in severely ill children with *P. falciparum *malaria [2-4,12], more accurate assessment of cardiac function in malaria is difficult to perform either because invasive monitoring is not indicated or because it is impractical in hospital settings of developing countries. Furthermore, no studies have been performed to date on patients with uncomplicated malaria. In this study, an innovative non-invasive method was used to assess cardiac output in travellers returning with *P. falciparum *malaria. In addition, cardiac enzymes and pro- and anti-inflammatory cytokines were analysed. Findings were compared in an unmatched case-control approach.

## Methods

### Study population

Between June 2007 and May 2008, patients hospitalized for *P. falciparum *malaria at the University Medical Centre Hamburg-Eppendorf, Section Tropical Medicine, were included. Patients above 18 years of age were eligible for participation if they had no known illness or hereditary malformation related to the heart influencing cardiac function and no known lung disease. Patients were clinically examined, blood samples were obtained and cardiac function was assessed within 24 hours after hospital admission. *Plasmodium falciparum *malaria was defined as presence of asexual blood stage parasites and no evidence for other infectious diseases. Complicated malaria was defined according to national guidelines by the German Society for Tropical Medicine [13]. Briefly, complicated malaria is defined as the presence of at least one of the following criteria: i) impaired consciousness, ii) hypoglycaemia (blood glucose < 40 mg/dl), iii) clinical signs of circulatory collapse, iv) spontaneous bleeding, v) acidosis (base deficit > 8 mmol/l), vi) hyperbilirubinaemia/jaundice (bilirubin > 3 mg/dl), vii) anaemia (haemoglobin < 8 g/dl), viii) renal insufficiency (creatinine > 2.5 mg/dl), ix) hyperparasitaemia (> 5% parasitized red blood cells), and x) elevated transaminases (> 3x above upper limit). Antiparasitic treatment was started accordingly with either mefloquine, artemether-lumefantrine or a quinine-doxycyclin combination including usual supportive care as clinically indicated. In an unmatched case-control design, unrelated healthy German adults without clinical signs of ongoing infectious disease or known cardiac or pulmonary disease served as controls. Approval of the local ethics committee was obtained and written informed consent was obtained from each patient and control subject.

### Parasitology, blood chemistry

*Plasmodium falciparum *malaria was ascertained from Giemsa-stained thick and thin blood films. Routine laboratory parameters including differential blood count, creatinine, total bilirubin and lactate dehydrogenase (LDH) were measured at the central university laboratory according to standardized procedures. The analysis of the cardiac proteins N-terminal pro-brain natriuretic peptide (NT-proBNP), myoglobin, creatine kinase-muscle brain (CK-MB), and troponin T (TnT) was also performed as part of the routine panel at the central university laboratory. Heart-type fatty acid-binding protein (H-FABP) was determined by enzyme-linked immunosorbent assay (Abcam Ltd.^®^, Cambridge, UK). From all patients and controls, additional serum samples were obtained and stored at -80°C until further analysis of cytokines.

### Assessment of cardiac function

In addition to clinical symptoms and conditions defining severe malaria [1,13], cardiac function was assessed by a non-invasive method based on a single-alveolar lung model. This method has been evaluated in several independent investigations [e.g. 14, 15]. The principle of this method is based on re-breathing a gas mixture and calculating the pulmonary blood flow from the dilution of a soluble gas. It is assumed that (i) gases within alveolar and dead space air as well as the bag volume mix completely and instantaneously, (ii) instantaneous equilibrium of soluble gas between alveoli and blood and between alveoli and tissue occurs, respectively, (iii) constant pulmonary blood flow (PBF) and constant lung volume of lung tissues given, and (iv) mixed venous concentration of soluble gas throughout the re-breathing period is negligible. For the latter assumption, it is required that no re-circulation (shunt) occurs and repeated measurements are carried out after sufficient washout periods (e.g. 5 minutes). During the re-breathing period, the concentration of an insoluble gas (sulphur hexafluoride, SF_6_) decreases from the initial value in the bag (F_i_^0^) to a final equilibrium (F_i,eq_) value practically obtained after a few breaths. Since the volume of the re-breathing bag is known, the total ventilated volume can be determined from the dilution of the insoluble gas. The nearly instantaneous disappearance of the soluble gas nitrous oxide (N_2_O) is ascribed to solution of the gas in lung tissue. The disappearance rate is proportional to the pulmonary blood flow. Assuming a constant PBF and a constant total ventilated volume, the disappearance curve for the soluble gas describes a monoexponentially decreasing function of time since the rate of absorption is also proportional to the alveolar gas concentration. Pulmonary blood flow is then calculated. Cardiac output can be derived from pulmonary blood flow after correction for intrapulmonary shunt fraction. By including parameters like blood pressure, heart rate, peripheral oxygen saturation, weight and height, further parameters like stroke volume and systemic vascular resistance were calculated. To account for differences in height and weight, measurements for cardiac output (CO, [ml/min]), stroke volume (SV, [ml]) and systemic vascular resistance (SVR, [ mmHg/(l/min)]) were related to the body surface [m^2^] and respective cardiac-, stroke- and systemic vascular resistance indices (CI, SI, SVRI) were included into statistical analyses. Patients did not receive volume infusions shortly before or during the re-breathing measurements.

### Cytokine analysis

Sera of the patients were analysed for pro- and anti-inflammatory cytokines with a Cytokine Bead Array following manufacturer instructions (Becton Dickinson^® ^(BD) Biosciences, Heidelberg, Germany). The following cytokines were measured: IL-2, IL-6, TNF-α, IFN-γ (Th-1 response), IL-4, IL-10 (Th2-response), and IL-17A (Th-17 response). Briefly, phycoerythrin (PE)-conjugated (emission maximum 575 nm; FL2) beads with distinct PE fluorescence intensities which have been coated with capture antibodies specific for the various cytokines were mixed with serum enhancement buffer and recombinant cytokine standards (20-5000 pg/ml) or patients serum samples. After centrifugation at 200 *g *for 5 min the supernatants were aspirated and discarded. After incubation of the beads for 3 h with a PE detection reagent at room temperature, wash buffer was added and the suspension centrifuged as before. After discarding the supernatant the beads were resuspended in wash buffer for acquiring and analysis of the samples in a flow cytometer (FACS Calibur^®^, BD) applying BD CellQuest^® ^software.

### Statistics

The SPSS 17.0 software package (SPSS Inc.^® ^Chicago, IL, USA) was used for statistical analysis. Normal distribution was assessed by the Kolmogorov-Smirnov test. The independent sample t-test or the Mann-Whitney *U*-test were used to assess differences between cases and controls, as appropriate. Proportions were assessed by chi-square test or Fisher's exact test (two-tailed) as applicable. For correlation of cardiac function tests and cytokines Pearson correlation test was used. Statistical significance was considered a P < 0.05.

## Results

### Baseline characteristics

Cardiac function, serological and parasitological parameters were investigated in 28 patients with imported *P. falciparum *malaria. All patients screened within the study period were found to be eligible according to the study protocol. Eighteen patients were African migrants whose reason to travel was visiting friends and relatives in their home countries and 10 German expatriates/tourists. All patients originating from endemic countries had lived in Germany for at least two years prior to recent travel to endemic regions. Cases consisted of 21 patients with uncomplicated malaria and 7 cases of complicated malaria. Criteria for complicated malaria were hyperbilirubinaemia (> 3 mg/dl, 5 cases), acute renal insufficiency (creatinine > 2.5 mg/dl, 1 case), hyperparasitaemia (> 5% parasitized erythrocytes, 2 cases), and jaundice (1 case). Geometric mean parasite density (GMPD) was 4,449/μl in all 28 patients and 2,673/μl and 22,315/μl in uncomplicated and complicated malaria cases, respectively (p = 0.01). Demographic, clinical and biochemical characteristics of malaria cases and healthy controls are shown in Table 1.

**Table 1:** Demographic, clinical and biochemical characteristics of malaria cases on hospital admission and healthy controls

	Malaria cases	Uncomplicated cases	Complicated cases	Healthy controls	*P*-value*
	*n *= 28	*n *= 21	*n *= 7	*n *= 26	
Age (± SD)	44.0 (± 11,8)	42.1 (± 10.2)	49.3 (± 15.3)	31.2 (± 15,4)	0.001
Male sex (%)	17/28 (61)	13/21 (62)	4/7 (57)	10/26 (38)	0.1
Temperature, °C (± SD)	37.9 (± 1.0)	38.1 (± 1.0)	37.7 (± 1.1)	36.5 (± 0.4)	< 0.001
Respiratory rate,/min (± SD)	17 (± 4)	16 (± 4)	19 (± 5)	17 (± 1)	0.7
Haemoglobin, g/l (± SD)	12,8 (± 1.7)	12.7 (± 1.8)	13.0 (± 1.6)	13,6 (± 1.1)	0.07
Thrombocytes, bn/l (± SD)	64,8 (± 39,6)	73,4 (± 38,7)	40,4 (± 33,2)	252,3 (± 102,9)	< 0.001
LDH, U/l (± SD)	334 (± 113)	298 (± 97)	425 (± 100)	149 (± 23)	< 0.001
Total bilirubin, mg/dl (± SD)	2.7 (± 4.2)	1.3 (± 0.4)	5.9 (± 7.1)	0.6 (± 0.4)	0.02
Creatinine, mg/dl (± SD)	1.1 (± 0.5)	1,04 (± 0.4)	1.4 (± 0.6)	0.8 (± 0.1)	0.003

*comparison between malaria cases (all) and healthy controls (by Mann-Whitney *U-*test)

### Cardiac function

Mean arterial blood pressure was comparable with 86 mmHg and 84 mmHg in malaria cases and controls, respectively (P = 0.7). Heart rate was significantly higher in malaria patients than in controls (86/min versus 70/min, P < 0.001; Table 2). Both, cardiac index (CI) and stroke index (SI) were highly significantly reduced in malaria patients compared to controls (CI: 2.7 l/min/m^2 ^versus 3.4 l/min/m^2^, P < 0.001; SI 31.3 ml/m^2 ^versus 43.6 ml/m^2^, P < 0.001, respectively). The systemic vascular resistance index (SVRI), in contrast, was significantly elevated in malaria patients (cases: 32.6, controls 23.2, P < 0.001). Cardiac function parameters did not differ significantly between uncomplicated and complicated malaria cases (see Table 2).

**Table 2:** Cardiac function parameters of malaria cases and healthy controls

	Malaria cases	Uncomplicated cases	Complicated cases	Healthy controls	*P*-value*
	*n *= 28	*n *= 21	*n *= 7	*n *= 26	
MAP, mmHg (± SD)	86 (± 11)	86 (± 12)	86 (± 10)	84 (± 13)	0.7
Heart rate,/min (± SD)	86 (± 14)	87 (± 13)	83 (± 16)	70 (± 8)	< 0.001
CI, l/min/m^2 ^(± SD)	2.7 (± 0.9)	2.7 (± 0.9)	2.9 (± 0.6)	3.4 (± 0.3)	< 0.001
SI, ml/m^2 ^(± SD)	31.3 (± 8.2)	30.3 (± 8.7)	34.2 (± 6.3)	43.6 (± 4.9)	< 0.001
SVRI, mmHg m^2^/(l/min) (± SD)	32.6 (± 10.3)	33.8 (± 11.0)	29.2 (± 7.2)	23.2 (± 5.2)	< 0.001

MAP = mean arterial blood pressure; CI = cardiac index; SI = stroke index; SVRI = systemic vascular resistance index

*comparison between malaria cases (all) and healthy controls (by Mann-Whitney *U-*test)

### Cardiac enzymes

Findings on cardiac function were paralleled by differences in cardiac enzymes. All cardiac proteins except for TnT were about twice as high in malaria patients compared to controls albeit at borderline statistical significance for NT proBNP and myoglobin (Table 3). Logistic (multiple) regression analysis showed that cardiac proteins were elevated in malaria patients independent of creatinine levels. Levels of cardiac proteins were inconsistent in patients with uncomplicated and complicated malaria: While NT proBNP, myoglobin and H-FABP were highest in patients with complicated malaria the level of CK-MB in complicated malaria was comparable with the level in healthy controls.

**Table 3:** Cardiac proteins in malaria cases and healthy controls

	Malaria cases	Uncomplicated cases	Complicated cases	Healthy controls	*P*-value*
	*n *= 28	*n *= 21	*n *= 7	*n *= 26	
NT-proBNP, ng/ml (± SD)	139.3 (± 146.6)	117.7 (± 140.3)	231.1 (± 156.2)	60.4 (± 48.2)	0.07
CK-MB, ng/ml (± SD)	21.5 (± 36.9)	24.0 (± 40.7)	10.8 (± 6.1)	12.1 (± 10.3)	0.08
Myoglobin, mg/l (± SD)	43.6 (± 12.5)	42.9 (± 12.0)	45.4 (± 15.0)	27.8 (± 15.0)	< 0.001
TnT, ng/ml (± SD)	< 0.1 (na)	< 0.1 (na)	< 0.1 (na)	< 0.1 (na)	ns
H-FABP, ng/ml (± SD)	1.9 (± 1.1)	1.7 (± 1.0)	2.7 (± 1.0)	1.1 (± 1.6)	< 0.01

NT-proBNP = N-terminal pro-brain natriuretic peptide; CK-MB = creatine kinase-muscle brain; TnT = troponin T; H-FABP = heart-type fatty acid-binding protein

*comparison between malaria cases (all) and healthy controls (by Mann-Whitney *U-*test)

### Cytokine analysis

**Figure 1:**
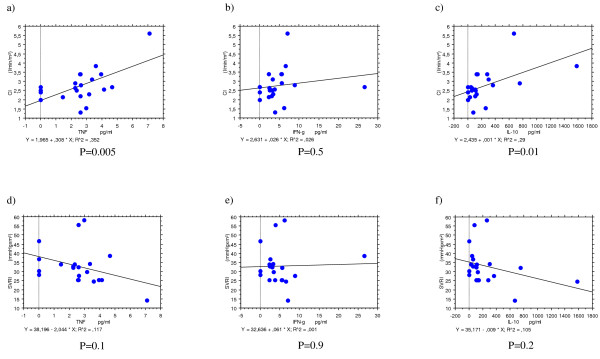
Correlation of host immune activation expressed by cytokine levels of TNF-α, IFN-γ and IL-10 with cardiac index (CI), a) – c), and systemic vascular resistance index (SVRI), d) – e).

To explore potential pathophysiologic causes for cardiac impairment in patients with malaria associations between cardiac function parameters and cardiac proteins with parasite densities and several cytokines representative for pro- and anti-inflammatory immune stimulation were assessed. Neither cardiac function (CI, SI) and systemic vascular resistance (SVRI) nor cardiac enzymes were associated with parasite levels (data not shown). Cytokine levels of malaria cases and controls as well as patients with uncomplicated and complicated malaria are summarized in Table 4. All pro- or anti-inflammatory cytokines assessed were markedly elevated in malaria patients compared to controls. All cytokines except for IFN-γ indicated a positive correlation with CI and a negative correlation with the SVRI albeit not always statistically significant (Figure 1). There was no association between CI, SI or SVRI with neither haemoglobin nor creatinine levels.

**Table 4:** Cardiac proteins in malaria cases and healthy controls

	Malaria cases	Uncomplicated cases	Complicated cases	Healthy controls	*P*-value*
	*n *= 28	*n *= 21	*n *= 7	*n *= 26	
IL-2, pg/ml (± SD)	1.3 (± 1.5)	1.1 (± 1.3)	2.2 (± 1.9)	0.5 (± 0.9)	0.04
IL-6, pg/ml (± SD)	33.8 (± 45.0)	36.8 (± 49.8)	21.0 (± 7.5)	5.0 (± 9.0)	< 0.001
TNF, pg/ml (± SD)	2.6 (± 1.7)	2.4 (± 1.8)	3.4 (± 1.0)	1.5 (± 0.7)	0.002
IFN-γ, pg/ml (± SD)	4.9 (± 5.5)	3.3 (± 2.2)	11.6 (± 10.1)	1.9 (± 0.7)	< 0.001
IL-17A, pg/ml (± SD)	35.7 (± 29.6)	26.9 (± 16.9)	72.9 (± 45.2)	21.0 (± 9.7)	0.02
IL-4, pg/ml (± SD)	2.7 (± 1.6)	2.4 (± 1.5)	3.9 (± 1.5)	1.3 (± 1.1)	0.001
IL-10, pg/ml (± SD)	246.4 (± 366.8)	256.2 (± 404.7)	204.4 (± 142.7)	2.0 (± 0.8)	< 0.001

UM = uncomplicated malaria; CM = complicated malaria

*comparison between malaria cases (all) and healthy controls (by Mann-Whitney *U-*test)

## Discussion

The results presented here show impaired myocardial function in patients with imported *P. falciparum *malaria. Cardiac output (CO) representing the blood volume pumped per minute was significantly decreased in malaria patients compared to healthy controls. The increase in heart rate, however, was not sufficient to compensate for the lower stroke volume in malaria patients. This finding is in line with recent publications supporting evidence for cardiac dysfunction in malaria patients [4,12].

Reasons for impaired cardiac function may be related to impaired pre- or postcardial circulatory parameters or result from myocardial dysfunction itself. In recent years, intravascular fluid depletion has been shown to be present in patients with severe malaria contributing to at least those pathophysiological processes related to impaired microcirculation [16]. In patients with malaria studied here, blood pressure was not affected in either direction. Moderate volume depletion may be present and contribute to reduced CO by reducing the pre-load. This pathophysiological background, however, does not seem to be the most prominent reason for reduced CO in the patient group. Except for malaria infection, all patients were otherwise healthy and well nourished. There were no differences in cardiac function parameters between complicated and uncomplicated malaria cases which may be due to the fact that comparatively broad criteria were used to define complicated malaria and none of the complicated cases was life-threatening. Volume depletion may become a more important issue in severe malaria in endemic countries: Children carrying the major burden of severe malaria are at high risk for rapid fluid loss due to relatively high body surface combined with high fever as well as additional risk factors like diarrhoea, high environmental temperatures, and possibly limited access to effective fluid uptake.

By the methodological approach used in this study, it was possible to calculate peripheral vascular resistance which was significantly increased in patients with malaria. This may indicate that in this patient group increased after-load might contribute to the decreased CO. Major pathophysiological processes typical for *P. falciparum *malaria are the parasite adhesion to the endothelium, rosetting, the sequestration of parasitized and unparasitized red blood cells (RBC) in peripheral small vessels, and the decreased deformability of RBCs resulting in impaired microcirculation and lactic acidosis [9,17-21]. The cytoadherence and rosetting properties of parasitized erythrocytes have been shown to significantly increase flow resistance in a recent numerical hemodynamic analysis [22]. It was not possible to assess the amount of sequestration in these malaria patients. Cardiac output, however, did not correlate with blood parasite levels. This does not rule out an association of increased after-load with parasite sequestration in the periphery as parasitaemia assessed from large central veins does not necessarily correlate with parasite numbers sequestered in small vessels and capillaries [23]. Interestingly, significantly elevated levels of d-dimer, a degradation product of cross-linked fibrin, were present in this patient group indicating increased fibrinolytic activity in malaria. D-dimer correlated positively with CO. Coagulation processes may contribute to both, thrombocytopenia and impaired microcirculation. Nevertheless, increased peripheral resistance due to parasite-related increased flow-resistance may not be sufficient to significantly affect cardiac function. It is likely that our findings on reduced cardiac output are based on several co-occurring and interdependent factors like reduced preload and impaired myocardial function.

Finally, malaria may affect cardiac function itself. NT-proBNP is a sensitive marker for impaired left ventricular (LV) function and is significantly elevated predominantly in severe malaria patients [2,3]. Parasite toxins or host immune mediators or both may have a suppressive effect on myocardial function. It has been shown that the plasmodial toxin glycosylphosphatidylinositol (GPI) augments apoptosis rates in cardiomyocyte culture [24,25]. On the other hand, the host immune reaction against malaria parasites involves pro- and anti-inflammatory cytokines as well as immune mediators like nitric oxide (NO). While pro-inflammatory cytokines and immune mediators have been shown to exert a suppressive effect on myocardial function, cardiovascular biomarkers have been reported as good prognostic markers for outcome in septic and critically ill patients [6,26]. Recent publications support myocardial suppression in malaria [27,28] although other evidence is still contradictory [29]. In addition, anti-malarial treatment may add to cardiotoxic effects [30].

To assess the influence of the host immune reaction, a series of pro- and anti-inflammatory cytokines was assessed. As anticipated, all cytokines assessed were significantly elevated in malaria patients compared to controls, especially IL-17A in complicated malaria (Table 4). Their association with cardiac function parameters, however, was inconsistent. Most pro- and anti-inflammatory cytokines except for IFN-γ were associated with increased cardiac output on the one hand and decreased peripheral vascular resistance on the other (see Figure 1). It has been described that pro-inflammatory cytokines like TNF-α have can impact myocardial function via negative-inotropic effects which may play a role in malaria patients [31]. The study design was not designed to explore the role of these cytokines any further or to discriminate between Th1-, Th2-, or Th17 cytokines with respect to cardiac function. If elevated cytokines represent activated host immunity in general, this seems to have an overall influence on cardiac function in malaria similar to the hyperdynamic but haemodynamically inefficient status described in bacterial sepsis [5-7,32,33]. In a subset of patients, however, pathogenic features characteristic for malaria resulting in impaired microcirculation may outweigh this effect related to unspecific host immune defence mechanisms. While high-output failure is typical for bacterial sepsis and high fluid substitution as been long established as life-saving therapy, pathophysiological processes appear to be more heterogenic in malaria leading to (mild) low-output failure at least in a subset of patients. Yet, findings could be interpreted in that moderate and careful volume substitution may be preferable over strict volume depletion in this highly febrile disease as it may not only improve cardiac output but also may have a beneficial effect on microcirculation, tissue perfusion and lactic acidosis. However, there is no evidence derived from randomised clinical trials that volume substitution improves mortality in severe malaria.

The present study has some limitations. A rather small patient population was assessed which increases the risk of type I errors. Furthermore, the study population was rather heterogeneous in that pathophysiological processes may differ in German travellers and African migrants. All African migrants, however, had lived in Germany for at least two years and persistent semi-immunity was unlikely to have a major influence on the findings presented here. Finally, one substantial limitation results from the unmatched control group despite the fact that respective cardiac function parameters were expressed as indices to correct for differences in body weight and height.

The innovative non-invasive methodological approach allowed the assessment of several dynamic functional parameters. As it is usually not indicated or possible to assess cardiovascular function by the gold standard of invasive thermodilution techniques our findings may contribute to the existing evidence on cardiovascular function in malaria patients. To obtain further information on the cardiovascular system in patients with malaria a larger scale trial on cardiac function in children with severe malaria is currently being completed in hyper-endemic regions of Ghana.

## Conclusions

Findings presented here support previous reports suggesting impaired cardiac function contributing to clinical manifestations in *P. falciparum *malaria. Reduced cardiac output is paralleled by increased systemic vascular resistance. Findings may be relevant for fluid management and should be further explored in endemic regions.

## Competing interests

The authors declare that they have no competing interests.

## Authors' contributions

Subjects were mainly recruited by JH with contributions by JPC, DW and SS. JPC and JH drafted the manuscript. NWB carried out the immunoassays. JPC and JH participated in the design of the study, PM and JPC performed the statistical analyses with major contributions from the other authors. JPC and GDB conceived of the study, and participated in its design and coordination and helped to draft the manuscript. All authors read and approved the final manuscript.
